# Tramadol Extended-Release for the Management of Pain due to Osteoarthritis

**DOI:** 10.1155/2013/245346

**Published:** 2013-09-04

**Authors:** Chiara Angeletti, Cristiana Guetti, Antonella Paladini, Giustino Varrassi

**Affiliations:** Anesthesiology and Pain Medicine, University of L'Aquila, Viale San Salvatore, Edificio 6, Coppito, 67100 L'Aquila, Italy

## Abstract

Current knowledge on pathogenesis of osteoarticular pain, as well as the consequent several, especially on the gastrointestinal, renal, and cardiovascular systems, side effects of NSAIDs, makes it difficult to perform an optimal management of this mixed typology of pain. This is especially observable in elderly patients, the most frequently affected by osteoarthritis (OA). Tramadol is an analgesic drug, the action of which has a twofold action. It has a weak affinity to mu opioid receptors and, at the same time, can result in inhibition of the reuptake of noradrenaline and serotonin in nociceptorial descending inhibitory control system. These two mechanisms, “opioidergic” and “nonopioidergic,” are the grounds for contrasting certain types of pain that are generally less responsive to opioids, such as neuropathic pain or mixed OA pain. The extended-release formulation of tramadol has good efficacy and tolerability and acts through a dosing schedule that allows a high level of patients compliance to therapies with a good recovery outcome for the patients' functional status.

## 1. Background

Pain is the most common symptom of osteoarthritis (OA), and, as pain levels rise, patients experience a reduced range of motion with a consequent increase of disability [1]. 

Pain and function limitations substantially reduce the life quality of people affected by OA. The treatment planning for OA is designed to essentially provide pain relief, to prevent from complications such as muscle atrophy or joint deformities, and to maintain and/or improve the functional status with the final aim to produce a sensible life quality improvement [2]. 

The effectiveness of pain relief not only may result in a reduction of the intensity of pain itself but can also lead to an improvement of life aspects that are strictly related to pain. As has been widely documented, chronic persistent pain can sensibly reduce the health-related quality of life, causing reduced sleep, interference with social/family relationships [3], activity of daily living and productivity, and increased anxiety and depression [4]. 

There is, therefore, a general need for optimized pharmacologic treatment strategies in patients with chronic/persistent pain due to OA. A management strategy for such patients also should require individualized therapies that are able to ensure a positive risk/benefit profile. It should also provide analgesia outcomes covering an extended period of time. Tramadol is a centrally acting synthetic analgesic with two mechanisms of action. It involves weak *μ*-opioid receptors agonism and inhibition of reuptake of both norepinephrine and serotonin with resulting in descending modulatory pain inhibition system reinforcement. Tramadol is available in several formulations and dosages, including immediate-release (IR) and once-daily extended-release (ER) forms. Several extended-release technologies are marketing with different pharmacokinetic characteristics (onset time; steady state time; plasma levels at 24 h; and *C*_max_) with different clinical implications.

Immediate-release formulation determines period of pain under treatment due to fluctuations of drugs plasma levels and frequent occurrence of adverse events. Reducing the frequent administration of the IR, it allows to obtain a major compliance to therapy with a more consistent round the clock pain relief. Another factor to take into consideration is that many patients often forget to take their next dose of IR medication until the pain returns, a phenomenon known as “chasing the pain.” Thus, ER formulation analgesics would be useful to treat OA chronic pain to avoid the “end of dose” pain episodes [5]. 

**Figure 1:**
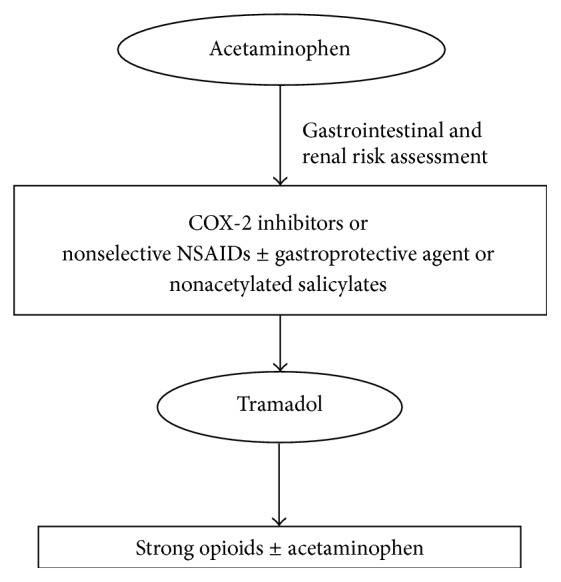
ACR, American College of Rheumatology guidelines for pharmacological management of OA [8].

In view of these considerations, tramadol ER formulations have been recommended in recent American and European guidelines for management of chronic OA pain (Figure 1) [6–8]. 

## 2. Clinical Pharmacology

### 2.1. Mechanism of Action

Tramadol (1RS, 2RS)-2-[(dimethylamino) methyl]-1-(3-methoxyphenyl)-cyclohexanol hydrochloride is a synthetic opioid of the aminocyclohexanol group, an analgesic with opioid agonist properties, and acting on noradrenalin and serotonin neurotransmission [9, 10]. 

Tramadol is a racemic mixture, (−)-tramadol is about 10 times more potent than (+)-tramadol in inhibiting noradrenalin uptake, and (+)-tramadol is about 4 times stronger than (−)-tramadol in inhibiting serotonin uptake. Both enantiomers act synergistically to improve analgesia without increasing the adverse effects [11]. 

Tramadol is a relatively weak *μ*-opioid agonist; its affinity for *μ*-opioid receptors was 6000, 60, and 10 times lower than that of morphine, dextropropoxyphene, and codeine, respectively [12]. The opioid component (agonist action at the supraspinally *μ*-opioid receptor) is primarily contributed by the *O*-desmethyl metabolite (M1) of tramadol [13]. 

Tramadol is metabolized by *O*-demethylation rapidly and widely. It has been suggested that tramadol is a prodrug, and M1 is important for the analgesic effects [14–16]. 

Analgesic effect of tramadol is not completely blocked by naloxone; in fact, experimental evidence seems to show a different mechanism involving nonopioid analgesic mechanism. Tramadol seems to inhibit ascending pain signaling covered by opioid component and amplification of descending pain modulatory system through nonopioid component.

Main tramadol nonopioid mechanisms consist in the inhibition of spinal neuronal reuptake and enhanced release of 5-HT and norepinephrine (NE) with consequent increase of extra neuronal concentration of these neurotransmitters [16]. 

In details tramadol seems to produce a dose-dependent and complete inhibition of locus ceruleus (LC) activity in vivo through alpha(2)-adrenoceptors. Moreover, this effect is modulated by the 5-HT system and particularly by 5-HT (1A) receptors [17]. 

Moreover, release of noradrenaline (NA) caused by (+)-M1 and the inhibition of the NA reuptake due to the action of (−)-M1 were indicated, resulting in a positive contribution to antinociception of the predicted increasing levels of NA [18]. 

Because tramadol increases 5-HT in the central nervous system, the serotonergic system has been suggested to be involved in tramadol analgesia [19, 20]. 

Conflicting results exist in the literature about the role of serotonergic receptor subtypes in systemic tramadol-induced antinociception. There are seven families of 5-HT receptors (5-HT1-7), and one of the most recently identified subtypes is the 5-HT7 receptor [21]. 

Immunocytochemical studies found that 5-HT7 receptors are localized in the superficial layers of the spinal cord dorsal horn receptors [22] and in the dorsolateral funiculus, which has been accepted as the main route for bulbospinal descending inhibition on the spinal transmission of nociceptive inputs, consistent with a predominant role of the 5-HT7 in the control of nociception [23]. 

Yanarates et al.'s findings suggest that the activation of descending serotonergic pathways and the spinal 5-HT7 receptors bindings are the main players in the antinociceptive and antihyperalgesic effects of tramadol and its metabolite M1 [24]. 

### 2.2. Pharmacokinetics

Bioavailability of tramadol hydrochloride ER 200 mg tablets, in healthy subjects, was approximately from 85 to 90%. Volume distribution of tramadol was 2.6 in male and 2.9 liters/kg in female subjects, after a 100 mg intravenous dose administration. The plasma protein binding of tramadol is approximately 20%, and it seems to be independent of concentration up to 10 mcg/mL [25]. 

Tramadol is mainly metabolized by the CYP enzyme system in the liver and excreted by the kidneys. Tramadol undergoes biotransformation in the liver, firstly by the phase I reactions (mainly* O*- and *N*-demethylation) and secondly by the phase II reactions (mainly conjugation of *O*- and *N*-demethylated compounds) [26].

**Figure 2:**
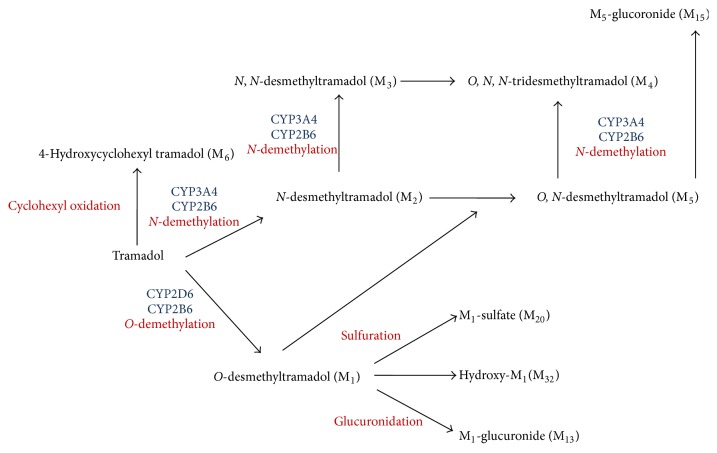
Metabolic pathways of tramadol [27–29].

In the phase I reactions, 11 metabolites and, in the phase II reactions, 12 metabolites are produced; the main metabolite is *O*-desmethyltramadol (M1) (Figure 2) [27]. 

Apart from *O*,*N*-didesmethyltramadol (M5, which exhibits weaker analgesic activity than M1), other metabolites are pharmacologically inactive. Tramadol is metabolized through CYP3A4 and CYP2B6 to *N*-desmethyltramadol (M2) and through CYP2D6 and CYP3A4 to M5 (Figure 2) [26–29]. The elimination half-life of tramadol is about 5-6 h and that of M1 is approximately 8 hours [30–32]. 

Upon oral administration of tramadol, about 90% of the drug is excreted by the kidneys and 10% with the feces. Approximately 30% of tramadol dose is excreted in the urine as unchanged drug, and the remaining 60% is eliminated as metabolites and is excreted as unidentified or unextractable metabolites [33].

Mean terminal plasma elimination half-lives of racemic tramadol and racemic M1 after administration of tramadol hydrochloride ER tablets are approximately 7.9 and 8.8 hours, respectively [34]. *O*-desmethyltramadol is released, after bioactivation, into the blood, enters the central nervous system, and activates *μ*-opioid receptors. Finally, *O*-desmethyltramadol is inactivated by glucuronidation in the liver, most probably by UGT2B7 [35]. 

The hepatic uptake of moderately hydrophobic tramadol and the more hydrophilic *O*-desmethyltramadol may require active, carrier-mediated transmembrane transport. OCT1 is the most abundantly expressed cellular drug transporter in the human liver and is a potential transporter for tramadol and *O*-desmethyltramadol [30–32]. 

### 2.3. Pharmacogenetics

Tramadol is a prodrug that requires bioactivation to *O*-desmethyltramadol for its analgesic activity. This bioactivation is catalyzed almost exclusively by the genetically polymorphic enzyme CYP2D6, and poor metabolizers of CYP2D6 substrates experience little, if any, analgesic effect from tramadol [36–38]. 

In ~10% of the Caucasian population, active CYP2D6 is totally absent, and bioactivation of tramadol is, therefore, not possible in these individuals [39]. 

However, as indicated by the large variation in pharmacokinetic and pharmacodynamic effects within subgroups defined by high, intermediate, or low CYP2D6 activity, CYP2D6 polymorphisms can explain only part of the high interindividual variation. This suggests that polymorphisms in other genes may also play a role [40, 41]. Common genetic polymorphisms cause high interindividual variability in OCT1 activity [42]. 

About 10% of the Caucasian population has substantially reduced or absent OCT1 activity [43]. 

Individuals with reduced or absent OCT1 activity showed higher blood concentrations of *O*-desmethyltramadol and stronger tramadol-induced miosis. The latter finding reflects opioidergic effects in the central nervous system and indicates that carriers of low OCT1 activity also had higher *O*-desmethyltramadol concentrations at drug target sites.

Individual response to tramadol is apparently not dependent on a single factor, but rather on multiple clinical and genetic factors [44]. 

Combined CYP2D6-OCT1 (and possibly OPRM1 and UGT2B7) pharmacogenetic analyses may be added to other methods of therapy individualization (such as patient controlled analgesia) in order to help optimize tramadol dosing. Tramadol is an important inhibitor of OCT1 that is a major transporter of *O*-desmethyltramadol; these findings may also help to understand and predict some drug-drug interactions [45]. 

OCT1 genotypes must be considered in tandem with CYP2D6 genotypes to improve our understanding of interindividual differences in tramadol pharmacokinetics and efficacy. Studies conducted in patients with postoperative pain demonstrated that patients devoid of CYP2D6 activity [27] (poor metabolizers, PMs) need approximately 30% higher tramadol doses than those with normal CYP2D6 activity (extensive metabolizers (EMs)) [39]. 

Genotyping is helpful in patients with duplication of the CYP2D6 gene (ultrarapid metabolizers (Ums)) as these patients are at greater risk to develop adverse effects to tramadol [46]. 

There were found an increased pain threshold and pain tolerance and a stronger miosis after tramadol infusion in UMs compared with EMs [40]. 

Duplication or multiduplication of the CYPD6 gene is associated with an ultrarapid metabolism of some compounds. Ultrarapid metabolizers (UMs) may experience either a lack of efficacy if the parent compound is responsible for the therapeutic effect of a given drug or very intense therapeutic effects associated with the production of an excessive amount of active metabolite(s) that may also be responsible for intense adverse effects [27]. Almost 50% of the UM group experienced nausea compared with 9% of the EM group. UMs were more sensitive to tramadol than EMs. Tramadol frequently causes adverse effects in southern European and northern African populations with a high proportion of UMs. 

The incidence of UMs is low in northern (1-2%), middle Europe, North America (4-5%), and Asia (0.5–2.5%) but is significantly higher in Mediterranean (7–12%), Saudi-Arabian (21%), and Ethiopian (29%) populations [40]. 

## 3. Use in Specific Populations

Patients with renal impairment (creatinine clearance: 79 mL/min) show a decreased excretion of tramadol and M1 in comparison to healthy individuals with normal renal function (creatinine clearance: 1 100 mL/min) [47]. 

Decreased rate and extent of excretion of tramadol and M1 were observed in patients with impaired renal function taking tramadol IR formulation. No studies have been performed on patients with renal impairment receiving tramadol ER formulations. It is recommended not to use tramadol ER formulations in patients with severe renal impairment (creatinine clearance less than 30 mL/min) [34]. 

Patients with advanced cirrhosis or hepatic failure showed a reduction of tramadol and M1 metabolism after tramadol IR administration, and this is evident through a larger area under the concentration time curve (AUC) for tramadol and longer mean tramadol and M1 elimination half-lives (13 hours for tramadol and 19 hours for M1). No data are available for tramadol ER used in patients with hepatic impairment. It is recommended not to use tramadol ER formulations in patients with hepatic impairment because limited availability of effective dose of tramadol ER does not permit the right dosing flexibility required for safe use in these patients [34]. In patients with advanced cirrhosis, there is a decrease in tramadol metabolism with a concomitant decrease in hepatic clearance and a rise in the blood serum levels. In these patients a 2.5-fold increase in the elimination half-life is observed [46–48]. 

In healthy elderly (aged 65–75), plasma concentrations and elimination half-lives after administration of an IR tramadol dose showed comparable values observed in healthy subjects less than 65 years of age. In elderly over 75 years, it was observed elevated mean maximum plasma concentrations (208 versus 162 ng/mL) and prolonged mean elimination half-life (7 versus 6 hours) compared to healthy elderly aged 65–75. It is an adequate adjustment of a daily dose for patients order than 75 years old. 

Tramadol has shown good profile of efficacy and was well tolerated in studies of elderly patients with chronic pain of various aetiologies [49]. 

In patients aged >65, tramadol should be started at the lowest possible dosage and incremented slowly to the efficacy dose. This effort of individualizing pain therapy is strongly desirable with tramadol because older patients are more likely to have augmented risk of renal or hepatic impairment and increased likelihood of comorbidity and concomitant pharmacotherapy. For this reason like other opioids, tramadol may play a crucial role as pain killer but with a increased potential risk of AEs onset in older patients [5]. 

It was found that after a 100 mg IV dose of tramadol, plasma clearance was 6.4 mL/min/kg in males and 5.7 mL/min/kg in females, and following a single oral dose of tramadol IR, females had a 12% higher peak tramadol concentration and a 35% higher area under the concentration-time curve compared to males [34]. 

In the specific multimodal pediatric pain treatment, one of the potential options should be tramadol both in postoperative and in chronic pain [50]. 

Based on published studies on pediatric patients, this drug has shown a good degree of efficacy and safety in postoperative setting, but also in dental and in other pain conditions [49–52].

These clinical experiences seem to indicate tramadol as a very promising drug in pediatric pain approach, because it has an analgesic potency as close as possible to NSAIDs and opioids, with lowest risk of respiratory depression [53] or common NSAIDs' adverse effects such as bleeding tendency, renal impairment, and aggravate asthma [54]. 

Several formulations of tramadol such as tablets and drops make its administration simpler and noninvasive route. This could permit even a long-term treatment in children, although further clarification is required in this field.

## 4. Drug Interactions

Tramadol metabolism through the CYP2D6 enzyme of CYP in the liver can be a reason for possible interactions with drugs that inhibit this enzyme [55]. 

It was observed after administration of tramadol IR formulations in healthy subjects that concentrations of tramadol were 20% higher in “poor metabolizers” compared to “extensive metabolizers,” while M1 concentrations were 40% lower. This applies to two commonly used drugs, that is, cimetidine and ranitidine. Combination of tramadol with selective serotonin reuptake inhibitors (SSRIs; fluoxetine, paroxetine, and to a lesser extent sertraline) inhibits CYP2D6 and may cause the serotonin syndrome because SSRIs, apart from inhibiting tramadol metabolism, increase the level of serotonin in the CNS; they should not be coadministered with tramadol. The serotonin syndrome may also appear with concomitant administration of monoamine oxidase inhibitors, olanzapine, risperidone, and venlafaxine [56]. 

On the other hand, mianserin and mirtazapine do not inhibit CYP2D6, but they are substrates of this enzyme [57]. 

Inhibition of tramadol metabolism may attenuate analgesia because (+)-M1 has significant opioid analgesic activity. Concomitant administration of tramadol ER with quinidine, a selective inhibitor of CYP2D6, can lead to increased concentrations of tramadol and reduced concentrations of M1.

Controversies regarding attenuation of tramadol analgesia caused by concomitant administration of ondansetron (a selective antagonist of the type 3 serotonin, 5HT 3, receptor) as it blocks spinal 5HT 3 receptors and competitively inhibits CYP2D6 are documented [58, 59]. 

Tramadol is also metabolized by CYP3A4. Administration of CYP3A4 inhibitors, such as ketoconazole and erythromycin, or inducers, such as rifampin and St. John's Wort, with tramadol ER may affect the metabolism of tramadol leading to altered tramadol exposure.

Tramadol analgesia is impaired by concomitant administration of carbamazepine, a CYP3A4 inducer, due to the acceleration of tramadol and M1 metabolism and increases in tramadol metabolism [60]. 

Concomitant administration of tricyclic antidepressants increases the risk of seizures. Tramadol should be avoided in patients with a history of epilepsy. However, tramadol administered alone does not influence the possibility of fits [61]. 

Rare reports of digoxin toxicity and changes in warfarin effects have been reported (with elevation of prothrombin times) in patients who use these drugs concomitantly with tramadol; however, the mechanism of interaction in these instances remains unclear [62]. 

An exhaustive list of potential drug interactions with tramadol is provided in Tables 4 and 5 [63, 64]. 

## 5. Tolerability and Safety of Tramadol ER

The most common AEs reported across all tramadol formulations were GI (nausea, constipation, and vomiting) and CNS-related events (dizziness, somnolence, and headache). 

Recently Langley et al. reviewed the safety profile of tramadol hydrochloride (tramadol) in the treatment of chronic osteoarthritis pain, with specific reference to the incidence of adverse events (AEs) reported in large clinical trials [65]. 

Most AEs were mild to moderate in severity and occurred more commonly during initial treatment than during maintenance treatment.

The overall AEs rates and the rates of selected GI and CNS AEs appeared to be dose dependent in fixed-dose studies [66, 67]. 

Differences in the rates of gastrointestinal and central nervous system AEs were seen between long-acting and immediate-release tramadol formulations (Table 1).

Most drug-related AEs were reported during the first 4 weeks of treatment, after which there was found a substantial decline in the incidence of new AEs. A higher incidence of AEs during initial exposure to tramadol than during continued therapy also was seen in the comparative study of tramadol OAD and tramadol SR. The mean time onset of the most commonly reported AEs was 13 days, with a median duration of 18 days [68]. 

The most common adverse events in a study of women taking tramadol ER 100, 200, or 300 mg were nausea, dizziness, vomiting, constipation, somnolence, pruritus, dry mouth, increased sweating, and fatigue [69]. 

Actions of different tramadol formulations are biologically similar; differences in pharmacokinetics, drug-release patterns, and availability may influence the incidence of AEs associated with tramadol. A study by Gana et al. [66] was reanalyzed by Vorsanger et al. [70], according to age stratification (younger < 65 years or elderly > 65 years). In this post hoc analysis, elderly patients were significantly more likely than younger patients to report constipation (27.5% versus 16.8% *P* < 0.001), fatigue (8.6% versus 4.3% *P* = 0.016), and anorexia (5.9% versus 2.6% *P* = 0.028).

Respiratory depression is rare in the chronic use of tramadol. Respiratory depression is connected with the opioid mode of tramadol action so if it does occur, naloxone should be administered intravenously. During tramadol treatment, CO_2_ sensitivity at *μ*-opioid receptors in the brainstem is decreased, but ventilatory response is not depressed [71]. 

However, respiratory depression can be observed during tramadol treatment in patients with cancer pain and renal impairment [72]. 

This is associated with the accumulation of the active metabolite (M1), which has a longer elimination half-life than the parent compound and binds to *μ*-opioid receptors.

Respiratory depression in a patient treated with tramadol for postoperative pain due to resection of renal carcinoma, with renal impairment (creatinine clearance 30 mL/min) and with UM genotype, was depicted. It is not appropriate recommending tramadol administration in UM genotype patients or with renal impairment [46]. 

Tramadol use has been associated with seizures. Some patients predisposed have an increased risk of seizures with tramadol use, which increases with doses above the recommended dose range (400 mg). Concomitant administration of tricyclic antidepressants, SSRI, neuroleptics, MAOIs, bupropion, opioids, and other drugs that reduce the seizure threshold, increases the risk of seizures. Tramadol should be avoided in patients with a history of epilepsy or with a recognized risk of seizure (head trauma, metabolic disorder, alcohol abuse, CNS infections, and drugs withdrawal) [27]. 

The development of a potentially life-threatening serotonin syndrome (SS) may occur with the use of tramadol products, particularly with concomitant use of serotonergic drugs such as SSRIs, SNRIs, TCAs, MAOIs, and triptans, with drugs which impair metabolism of serotonin (including MAOIs) and with drugs which impair metabolism of tramadol (CYP2D6 and CYP3A4 inhibitors).

Serotonin syndrome (SS) is a potentially lethal event caused by excessive serotonergic agonism of serotonin receptors in central and peripheral nervous system. SS may develop as a result of increased serotonin synthesis, decreased serotonin metabolism, increased serotonin release, inhibition of serotonin reuptake (e.g., SSRIs), and/or direct agonism of serotonin receptors. Syndrome is often a result of a prescription drug, overdose of causative drugs, and/or complex interactions among several drugs [73]. 

The most important clinical manifestations are characterized by

- neuromuscular hyperactivity (e.g., tremor, clonus, myoclonus, hyperreflexia, incoordination, and rigidity);
- autonomic hyperactivity (e.g., diaphoresis, fever, tachycardia, tachypnea labile blood pressure, and hyperthermia);
- altered mental status (e.g., agitation, confusion, hallucinations, and coma) and/or gastrointestinal symptoms (nausea, vomiting, and diarrhea) [74].

Like to the risk of seizures, SS may occur with tramadol monotherapy but appears to be more common following either excessive use/overdose or with the coadministration of other medications, particularly antidepressants. 

With regard to the concomitant administration of tramadol and antidepressant and their relative interaction, the SS has been described with fluoxetine [75], sertraline [76], paroxetine [77], citalopram [78], fluvoxamine [79], venlafaxine [80], trazodone [81], TCAs and monoamine oxidase inhibitors, olanzapine, and risperidone [82]. 

In addition, Gnanadesigan et al. [83] reported four cases of SS among residents in a long-term care facility, all who were prescribed tramadol in combination with either SSRIs or mirtazapine [84]. 

Recently Tashakori and Afshari [85] described in a case series tramadol overdose with clinical manifestations of potential serotonin syndrome in tramadol overdose. Previously was described by Takeshita in a single case of toxic overdose with tramadol resulted in features suggestive of serotonin syndrome [86]. Several investigations suggest that abuse potential of tramadol is relatively low. Opioid analgesics use is accompanied by concerns regarding the potential for abuse, dependence, diversion, misuse, addiction, tolerance, and withdrawal. In contrast, short-acting tramadol has been shown to have a low potential for abuse [87]. 

The reported abuse rate for all tramadol preparations was estimated in 0.05%, or fewer than 0.5–1 cases/100,000 [87], and over 95% of these cases involved patients with a history of alcohol and/or substance abuse. Actually rates of abuse/dependence with the ER tramadol formulation are being monitored [88]. 

Withdrawal has been reported after abrupt discontinuation and in some cases after reduction of tramadol doses. Classic withdrawal symptoms related to opioids use were observed (abdominal cramps, anxiety, depression, diarrhea, goose flesh, insomnia, nausea, lacrimation, restless, and sweating). In about 12% of cases, withdrawal symptoms were described as atypical with severe anxiety, panic attacks, confusion, paranoia, hallucinations, and paresthesia [71]. 

## 6. Extended-Release Formulations

Extended-release (ER) tramadol provides prolonged, more consistent plasma concentrations of drug compared with immediate-release agents, and it concretizes in minor fluctuations that could contribute to end-of-dose breakthrough pain.

Sustained-release formulations have been developed in order to deliver drug in a more controlled, safe, and prolonged manner, with better control of pain and fewer sleep interruptions. It finally resulted in higher quality of life and increased compliance because of simplified dosing regimen, minimizing adverse effects associated with uncontrolled drugs peak plasma levels [89]. 

Immediate-release (IR) tramadol taken orally, in healthy volunteers, has half-life of about 5-6 hours. Immediate release tramadol has to be assumed several times per day to achieve pain relief resulting in peak and troughs of plasma levels compared with extended-release formulations [90]. 

In Table 2 are summarized the different technology chemical methods used in extended released formulations commercially available. Several of this formulation are available in many dosage (100, 200, 300, and 400 mg) [89, 91]. 

Tramadol ER formulations are indicated for the management of moderate to moderately severe chronic pain in adult patients (aged >18) who require around-the-clock treatment of their pain for an extended period of time.

Tramadol ER should be initiated at a dose of 100 mg once daily and titrated upward as necessary in 100 mg increments every 5 days to the point of pain relief and depending on tolerability to a maximum of 300 mg/day. Barkin suggests minidosing of tramadol IR (50 mg) every 6 hours in selected patients who require a more narrow dosage range due to potential adverse events [63]. 

Patients who are receiving tramadol IR can be switched to tramadol ER doses across the full range of available dosage strengths [92]. If patient's total daily dose of tramadol IR is 200 mg or 300 mg, it can be switched to the same total daily dosage of tramadol ER, (it is not necessary to start with a 100 mg daily dose) and then individualized to the needs of the patient. In Table 3 are illustrated the conversion dosages from short-acting tramadol to extended-release formulations [92]. 

Hernandez-Lopez et al. investigated the rate and extent of tramadol bioavailability of the extended-release tramadol once-daily administration and found that were not affected by the time point of administration [93]. Total and maximum exposure of the product was bioequivalent after the intake in the morning and at night. Thus, the time point of administration may be adjusted to the patient's needs, an optimized way for treatment of different pain conditions, for example, where the pain maximum symptoms occur in the morning (rheumatoid arthritis), late in the afternoon (OA), or in the night (neuropathic pain) without any significant change in the in vivo performance [94]. 

**Figure 3:**
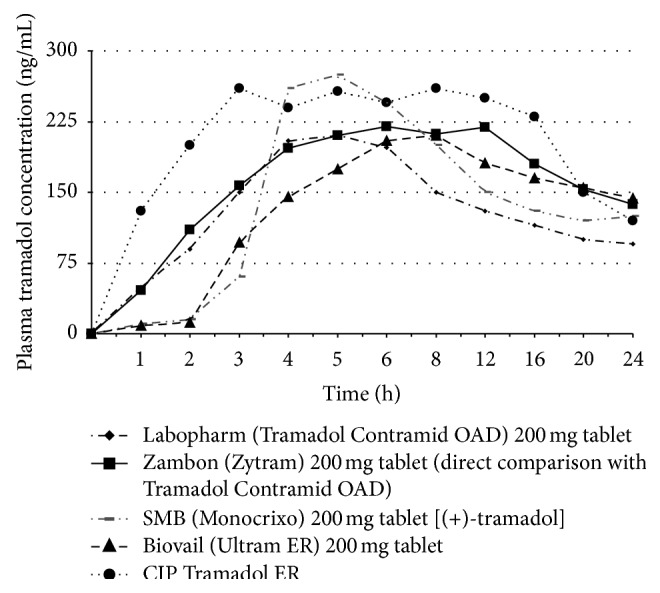
Mean plasma tramadol concentrations following single dose administration of ER formulation available in commerce [95–98].

A comparison of mean plasma tramadol concentrations following single-dose administration between five products using different chemical released technology is illustrated in Figure 3 [95–98]. 

## 7. Tramadol Extended-Release in OA

Several studies confirm the analgesic efficacy and safety of once-daily tramadol ER in osteoarthritis pain, the most important of which are reviewed here. In a double-blind, placebo-controlled-group study, which lasted 12 weeks, 246 patients with OA of the knee were randomized to tramadol ER or placebo when pain at the index knee joint reached > or =40 mm (0–100 mm VAS). Tramadol ER was started at 100 mg OAD but was allowed to increase to 200 mg OAD by the end of 1 week of treatment. After this first step, further augment to tramadol 300 mg or 400 mg QD were admitted. The results of Arthritis Pain Intensity VAS, over 12 weeks, show that pain relief with tramadol ER is better than placebo (least squares mean change from baseline: 30.4 mm versus 17.7 mm, *P* < 0.001). Already at week 1, significant differences from placebo were clear in terms of analgesia, stiffness, physical function, global status, and sleep. This experience confirms that tramadol ER is associated with statistically significant improvement in pain and physical function subscales of the Western Ontario and McMaster Universities (WOMAC) Osteoarthritis Index [99]. 

Another 12-week, randomized, double-blind, placebo-controlled-group, and multicenter trial was conducted by Gana et al., in 2006, including 1020 adults with osteoarthritis of the knee or hip. The patients with baseline pain intensity ≥40 on a 100 mm on pain visual analog scale (0 = no pain, 100 = extreme pain) were randomized to receive placebo or tramadol ER increased up to four levels of dosage, from 100, 200, and 300 up to 400 mg once daily. The WOMAC Osteoarthritis Index pain and physical function subscales showed that tramadol ER was significantly superior to placebo, overall (*P* ≤ 0.021) and for each dose (*P* ≤ 0.050). Tramadol ER 100–300 mg once daily provided significant pain relief and improved physical function, rather than tramadol ER 400 mg that determined the most of adverse events (e.g., constipation, dizziness, nausea, somnolence, and headache) [66]. 

Vorsanger et al. designed a randomized, double-blind, and placebo-controlled study, in 317 geriatric OA patients (65 years or older) to observe the improvement of Western Ontario and McMaster Universities (WOMAC) Osteoarthritis Index and in pain-related sleep parameters. This post hoc analysis suggests that tramadol ER 300 mg is the best dosage to improve pain (*P* < 0.05), pain-related sleep effects (*P* < 0.05), and physical function (*P* < 0.05), with optimal profile of efficacy and safety, in this frail populations also [70]. 

552 patients, between 40 and 75 years old, with pain associated with OA of the knee, were randomized in a placebo-controlled study with fixed dose of tramadol from 100 mg up to 300 mg, maintaining the titrate for 12 weeks. The high percentage of patients satisfaction for treatment efficacy (in 75%–80% of cases) at the dosage of 200 mg and 300 mg demonstrates that these therapeutical options are well tolerated and accepted to patients; furthermore, both formulations positively affect the WOMAC pain score, with 46% of improvement for 300 mg and 43% for 200 mg, respectively [67]. 

Another category with particular characteristic of pain is women. In a two parallel, placebo-controlled phase III clinical trials, efficacy and safety of tramadol ER were analyzed, 100, 200, and 300 mg daily, for up to 12 weeks compared with placebo in 685 women with moderate-to-severe pain due to osteoarthritis of the knee [68, 69]. A time-weighted analysis showed statistically significant improvements over placebo for all the WOMAC subscale scores of pain across all three dosages. All doses of tramadol ER once daily were more effective than placebo (*P* < 0,009, *P* < 0,034, and *P* < 0,043 for tramadol 100 mg, 200 mg, and 300 mg, resp.) on WOMAC subscale for physical function. The present study confirms that tramadol is effective for the management of painful osteoarthritis in women [69]. 

In 2008, a study was published that compares the efficacy and safety of tramadol 150 mg daily with placebo, and tramadol CR was titrated weekly to 200 mg, 300 mg, or a maximum of 400 mg once daily in patients with painful osteoarthritis. After four weeks, patients crossed over to the alternate treatment for another four weeks. Outcome measures included Arthritis Pain Intensity Visual Analogue Scale (VAS), Western Ontario and McMaster Universities Arthritis Scale (WOMAC) pain, Physical Function VAS subscales, Patient and Physician Global Assessment of Therapy, and sleep. Seventy-seven of 100 patients were randomized. All efficacy outcome measures favored tramadol CR over placebo. On the primary outcome variable VAS score is significantly lower than placebo (37.4 ± 23.9 versus 45.1 ± 24.3, *P* = 0.0009). and then individualized Treatment with tramadol CR results in statistically significant and clinically important and sustained improvements in pain, physical function, global status, and sleep in patients with chronic pain for up to six months, compared to placebo [100]. 

Recently a 12-week, multicenter, randomized, double-blind, placebo-controlled, and dose-ranging trial assessed tramadol ER (extended-release tramadol) in the management of knee and/or hip osteoarthritis. Pain intensity in the randomized patients was 40% on a 100 mm visual analog scale (0 = no pain, 100 = extreme pain), and they were assigned to assume once-daily tramadol ER 100 mg (*n* = 201), 200 mg (*n* = 199), or 300 mg (*n* = 199), celecoxib 200 mg (*n* = 202; to test model sensitivity), or placebo (*n* = 200). The most important efficacy variables are observed on the basis of WOMAC pain subscale, WOMAC physical function subscale, and patient global assessment of disease activity. With tramadol ER of 300 mg, patients obtained important global assessment scores compared with placebo (*P* < 0.05), but the other results are poor. Tramadol ER 200 and 100 mg do not improve symptoms and functions with respect to placebo for the coprimary efficacy variables. In this study, tramadol ER 300 mg determined a good improvement in severe painful osteoarthritis of the hip or knee compared to placebo [101]. 

Many patients with chronic pain due to OA suffer from sleep disturbances, some of which are pain related. Common sleep disturbances include difficulties in falling asleep, early awakening, and poor sleep quality. Wilcox et al. [102] reported in patient older than 65 year with chronic OA knee pain problems in sleep onset experienced in 31% of patient, sleep maintenance, and weekly early morning awakenings, respectively, in 81% and 51% of cases. 

Improvement in PRSDs in patients with chronic pain due to OA is recognized as a clinically important outcome; hence, sleep assessment should be included in the evaluation of analgesic efficacy and in overall pain management in patients with chronic pain. Florete et al., in a recent post hoc analysis of two 12-week, double-blind, placebo-controlled, randomized, and parallel-group studies, examined the effects of extended-release tramadol on reducing pain-related sleep disturbances (PRSDs) in patients with OA of the knee or hip. PRSDs were evaluated using the chronic pain sleep inventory (CPSI). In the first week, all groups receiving tramadol ER obtained significant better scores of sleep quality parameters (all *P* ≤ 0.022) comparing with placebo. For tramadol doses of 200 mg and 300 mg, quality of sleep improving was observed from the first week, while doses of 100 mg showed an improvement of sleep quality parameters in the third week of treatment (all *P* ≤ 0.046). This post hoc analysis shows that a reduction in pain was associated with a significant reduction in PRSDs due to OA [103]. 

## 8. Conclusions and Future Perspective 

In the past 30 years, the treatment guidelines for OA chronic pain suggested a multistage treatment algorithm based on increasing analgesic potency for greater efficacy and tolerability (Figure 1). Tramadol is recommended in those patients where use of oral nonselective NSAIDs (aspirin, naproxen, and ibuprofen) and COX-2 selective inhibitors can be problematic (e.g., in patients with history or increased risk of stroke or comorbid cardiac, gastrointestinal, or renal conditions that may be exacerbated by the use of these compounds) [63]. 

Tramadol ER, either as monotherapy or in combination with anti-inflammatory agents, could reduce the need for NSAID/COX-2 treatment rotations in patients with chronic/persistent pain. In fact, NSAIDs drug use is associated with a ceiling effect, a dosage beyond which efficacy is not increased [104]. Patients are often encouraged to switch to a different NSAID or a COX-2 inhibitor once they no longer experience pain relief with a certain agent [104]. 

NSAIDs have been associated with risks of gastrointestinal [105], cardiovascular [106], renal [107], haematological [108], and, less commonly, hepatic AEs [109].

NSAIDs are also associated to bleeding, fluid retention, hyperkalemia, peripheral oedema, increase in blood pressure, and acute renal failure; therefore, they should not be used in patients with a history of or risk factors for gastrointestinal ulceration, renal impairment, cardiovascular arrhythmias, myocardial infarction, or congestive heart failure [110]. 

COX-2 selective inhibitors had a more favorable gastrointestinal side effect profile than nonselective NSAIDs but are associated with an increased risk of serious cardiovascular thrombotic events, myocardial infarction (MI), and stroke [111–113]. Finally, NSAIDs may provoke reaction of hypersensitivity in atopic patient (asthma, nasal polyps, and rhinitis).

ER formulations can be particularly appropriate for long-term pain management in patients with multifactorial pain, such as OA. Tramadol showed a lower potential abuse than other centrally acting analgesics. An optimized treatment strategy could postpone and/or prescribe lower doses of traditional scheduled opioid analgesics. The low potential rate of abuse showed by tramadol is particularly important in view of recent alarming escalation use and related events of therapeutic opioids. Recently it has been illustrated that opioid analgesics are now responsible for more deaths than the number of deaths from both suicide and motor vehicle crashes or deaths from cocaine and heroin combined. A significant relationship was described between sales of opioid pain relievers and deaths [114]. 

Differences revealed on tolerability, pharmacokinetic, and pharmacological features of different ER tramadol formulations may direct physicians towards optimal individualised treatment of chronic OA pain subjects with tramadol. It can help physicians to fix the best maximal dose selection for each patient.

In the near future (probably within 5–10 years), the aim of analgesic therapies will try to hit individual genetic susceptibility in response to individual drug treatments. We could be able to individualise therapy for each patient. The better drug, for our clinical vision, is an improved formulation that is more suitable in order to avoid side effects and interactions and be able to draw the best possible effectiveness.

## Figures and Tables

**Table 1:** Reported adverse events ranges and discontinuation rates within each tramadol formulation [65].

Adverse event	IR 200–400 mg/day (4–6 doses/day)	CR 100–400 mg/day (QD)	SR 100–400 mg/day (QD or BID)	OAD 100–400 mg/day (QD)	ER 100–400 mg/day (QD)
Dizziness	6.8–17	5.3–24	8.1–36	9.7–25.6	16.8–33
Somnolence	6.8–28	15–37.2	11.7–21.4	6.7–30.2	8–20.3
Headache	5–18.5	2.1–22	4.5–17.7	<5–12.6	14.4–15.8
Nausea	17.5–53.7	16.2–42.6	22.5–34	11.3–32.6	14.9–25.7
Constipation	3.4–45	17.1–23.8	9.9–30.2	10.2–34	12.9–29.7
Vomiting	5–18.5	4.3–19	14.4–17.1	<5–14.8	5.4–9.4
Discontinuation Rate due to AEs	4.7–53.7	12.8–36.7	10.2–21.6	8.8–32.4	22.5–26.6

Frequencies of adverse events presentation are expressed as a percentage.

**Table 2:** Extended-release formulations technologies available in commerce [89, 91].

Formulation technology	Chemical description	Products
Contramid	Controlled release tablets consisting of cross-linked high-amylose starch with tramadol that forms semipermeable outer gel layer generating zero-order release. Compression coded tablet provides biphasic release (25% rapidly released; 75% released within 24 h)	Contramal UNO^®^, NOAX^®^ UNO, Dolpar^®^, Monalgic^®^, Monotramal^®^, Unitrama^®^, Tradorec^®^ XL, Tramadolor^®^, Unitrama^®^, Tidural^®^, Rysolt^®^, and Durotram^®^ XR

Smartcoat	Polymer diffusion-based film technology (drug released by osmosis). Semipermeable coating composed of water permeable film-forming polymer but not water soluble	Ultram^®^ ER

Mundipharma	Hydrophobic matrix system using hydrogenated castor oil	Dolzam^®^, Zitram^®^ XL, Tramagetic^®^, Travex^®^, Tiparol^®^ OD, Tradonal^®^ One, Zydol^®^ XL, and Zamadol^®^ 24 h

SMB Pellets	Pharmaceutical microgranules composed of a core containing tramadol and a porous membrane made of insoluble methacrylic polymers	Monocrixo^®^ LP, Tramium^®^, Tralodie^®^, T-Long^®^, and Dolodol^®^

Cipher technology	Capsules consisting of extended-release film-coated white beads and an immediate release tablet encapsulated (75% released within 7-8 h and 24 h; 25% rapidly released).	Cip Tramadol^®^ ER

**Table 3:** Conversion dosages from short-acting tramadol to extended-release formulations.

Total daily dose IR mg	Switch to ER tramadol once-daily dose, mg
125–175	100
200–275	200
300–375	300

**Table 4:** Specifics drugs interactions in concomitant tramadol administration.

Drugs	Drugs interaction/AEs	Actions/comments
Anticoagulants	Observed PT and INR increased. Reported extensive ecchymoses.	INR monitoring, caution is recommended.

Antifungals	Increased risk of seizures, serotonin syndrome.	Ketoconazole decreased tramadol clearance.

Carbamazepine	Risk of tramadol-associated seizures.	Concomitant use is not recommended. Increased metabolism of tramadol resulting in decreasing analgesic effect.

Cimetidine	Tramadol pharmacokinetics not altered.	

CNS depressants (alcohol, anesthetics, phenothiazines, sedatives, hypnotics, and opiates)	Risk of respiratory depression, CNS depression effects. Risk of fatal overdosage.	Caution is recommended; monitor patient closely, during treatment; use reduced dosage.

Digoxin	Rare episodes of digoxin toxicity are reported.	Caution is recommended; monitor patient closely, during treatment initiation and dosage escalation.

Linezolid	Increased risk of serotonin syndrome.	Caution is recommended; monitor patient closely, during treatment initiation and dosage escalation.

Lithium	Increased risk of serotonin syndrome.	Caution is recommended; monitor patient closely, during treatment initiation and dosage escalation.

Macrolides	Increased risk of seizures, serotonin syndrome.	Caution is recommended. Erythromycin decreased tramadol clearance.

MAO inhibitors	Increasing risk of seizures, serotonin syndrome.	Extreme caution is recommended.

Quinidine	Increased risk of seizures, serotonin syndrome.	Caution is recommended. Increased tramadol and decreases M1 concentrations.

Rifampin	Increased risk of seizures, serotonin syndrome.	Caution is recommended.

SNRIs	Increased risk of seizures, serotonin syndrome.	Caution is recommended; monitor patient closely, during treatment initiation and dosage escalation.

SSRIs	Increased risk of serotonin syndrome, seizures.	Caution is recommended monitor patient closely, during treatment initiation and dosage escalation. Fluoxetine and paroxetine (CYP2D6 inhibitors) may inhibit tramadol metabolism, with increasing tramadol and decreasing M1 concentrations.

Hypericum perforatum	Increased risk of serotonin syndrome, seizures.	Caution is recommended; monitor patient closely, during treatment initiation and dosage escalation.

Tricyclics	Increased risk of seizures, serotonin syndrome.	Caution is recommended monitor patient closely, during treatment initiation and dosage escalation. Amitriptyline may inhibit tramadol metabolism, with increasing tramadol and decreasing M1 concentrations.

Triptans	Increased risk of seizures, serotonin syndrome.	Caution is recommended; monitor patient closely, during treatment initiation and dosage escalation.

Modified from http://www.drugs.com/monograph/tramadol-hydrochloride.html.

**(a):** 

CYP2D6		
Substrates	Inhibitors	Inducers
Amphetamine	Amiodarone	Corticosteroids/ dexamethasone Delavirdine Protease inhibitors (indinavir, lopinavir/ritonavir, saquinavir)
Amitriptyline	Amitriptyline	
Aripiprazole	Bupropion	
Atomoxetine	Celecoxib	
Beta-blockers (selected)	Chlorpheniramine	
Carbamazepine	Chlorpromazine	
Carvedilol	Cinacalcet	
Chlorpheniramine	Chloroquine	
Chlorpromazine	Cimetidine	
Cinacalcet	Citalopram	
Clomipramine	Clomipramine	
Desipramine	Clemastine	
Dextromethorphan	Cocaine	
Doxazosin	Clozapine	
Flecainide	Desipramine	
Fluvoxamine	Diphenhydramine	
Haloperidol	Doxepin	
Hydrocodone	Doxorubicin	
Imipramine	Escitalopram	
Lidocaine	Duloxetine	
Metoclopramide	Fluoxetine	
Metoprolol	Fluphenazine	
Mexiletine	Fluvoxamine	
Nortriptyline	Haloperidol	
Ondansetron	Hydroxyzine	
Oxycodone	Lomustine	
Paroxetine	Methadone	
Perphenazine	Metoclopramide	
Primaquine	Mibefradil	
Promethazine	Nefazodone	
Risperidone	Norfloxacin	
Tamoxifen	Quinidine	
Thioridazine	Paroxetine	
Timolol	Perphenazine	
Tricyclic antidepressants	Propafenone	
Venlafaxine	Quinidine	
Yohimbine	Ranitidine	
	Ritonavir	
	Sertraline	
	Sertindole	
	Terbinafine	
	Thioridazine	
	Ticlopidine	
	Tricyclic antidepressants	
	Venlafaxine	
	Vinblastine	
	Vinorelbine	

**(b):** 

CYP2B6		
Substrates	Inhibitors	Inducers
Bupropion	Orphenadrine	Barbiturates
Cyclophosphamide	Thiotepa	Phenytoin
Efavirenz	Ticlopidine	Primidone
Ifosfamide		Rifampin
Meperidine		
Methadone		

**(c):** 

CYP3A4	
Inhibitors	Inducers
Amiodarone	Barbiturates
Aprepitant	Carbamazepine
Cannabinoids	Corticosteroids/dexamethasone
Chloramphenicol	Efavirenz
Cimetidine	Ethosux
Ciprofloxacin	Gluteth
Clarithromycin	Griseofulvin
Clomipramine	Modafinil
Clotrimazole	Nevirapine
Cyclosporine	Oxcarbazepine
Delavirdine	Phenylbutazone
Diltiazem	Phenytoin
Erythromycin	Primidone
Fluconazole	Rifabutin
Fluoxetine	Rifampin
Fluvoxamine	Sulfinpyrazone
Indinavir	Troglitazone
Itraconazole	
Ketoconazole	
Metronidazole	
Nefazodone	
Norfloxacin	
Norfluoxetine	
Omeprazole	
Paroxetine	
Propoxyphene	
Quinidine	
Ranitidine	
Ritonavir	
Sertraline	
Venlafaxine	
Verapamil	
Zafirlukast	
Zileuton	

## Appendices

### A. Specific Drugs Interactions in Concomitant Tramadol Administration

For more details see Table 4.

### B. Potential Tramadol Drug Interactions

For more details see Table 5.

### Practice Points

- Tramadol extended-release formulations (ER) have been recommended in recent American and European guidelines for management of chronic osteoarthritis (OA).
- Multimodal mechanism of action demonstrated by tramadol, opioid, and nonopioid components represents a synergic option for pain management.
- Tramadol ER formulation may reduce frequent administration of immediate-release formulation, pill burden, attenuation of drugs fluctuations plasma levels, and frequent occurrence of adverse events.
- Major compliance to therapy may be obtained with a more consistent round-the-clock pain relief.
- Several ER technologies are available with different pharmacokinetic characteristics and clinical implications.
- Tramadol metabolism depends on genetic highly polymorphic variability to be taken into account for clinically interindividual differences in tramadol pharmacokinetics and efficacy.
- Combination of tramadol with selective serotonin reuptake inhibitors (SSRIs) or simultaneous administration of monoamine oxidase inhibitors (MAO) may cause increase level of serotonin in the CNS with development of serotonin syndrome (SS), a potentially life threatening adverse event.
- Common reported opioid-related adverse effects (AEs) were central-nervous-system- (CNS-) related events (dizziness, somnolence, and headache) and gastrointestinal (nausea, constipation, and vomiting). Adverse effects were mild to moderate in severity and occurred more commonly during initial phase of treatment.
- Tramadol ER may postpone the need for scheduled opioids, provide opioid-sparing effects, and reduce the requirement for NSAIDs.

## Abbreviations

AEs: Adverse effects
CYP: Cytochrome
CNS: Central nervous system
COX: Cyclooxygenase
CPSI: Chronic pain sleep inventory
CR: Controlled release
EMs: Extensive metabolizers
ER: Extended-release formulations
GI: Gastrointestinal
IR: Immediate release
LC: Locus coeruleus
MAO/MAOIs: Monoamine oxidase inhibitors
M1: *O*-desmethyl metabolite
NE: Norepinephrine
NSAIDs: Nonsteroidal anti-inflammatory drugs
OA: Osteoarthritis
OAD: Once a daily
OCT: Organic cation transporter
OPRM: Mu opioid receptor
PMs: Poor metabolizers
PRSDs: Pain-related sleep disturbances
SNRIs: Serotonin-norepinephrine reuptake inhibitors
SS: Serotonin syndrome
SSRIs: Serotonin reuptake inhibitors
TCAs: Tricyclic antidepressants
UGT: UDP-glucuronosyl transferase
UMs: Ultrarapid metabolizers
VAS: Visual analogue scale.
